# Protective effect of andrographolide against ulcerative colitis by activating Nrf2/HO-1 mediated antioxidant response

**DOI:** 10.3389/fphar.2024.1424219

**Published:** 2024-07-29

**Authors:** Long Shu, Hangjie Fu, Aiwen Pi, Yuliang Feng, Hui Dong, Caijuan Si, Songtao Li, Feiye Zhu, Peifen Zheng, Qin Zhu

**Affiliations:** ^1^ Department of Clinical Nutrition, Zhejiang Hospital, Hangzhou, China; ^2^ School of Public Health, Zhejiang Chinese Medical University, Hangzhou, China; ^3^ Department of Digestion, Zhejiang Hospital, Hangzhou, China; ^4^ Academy of Chinese Medical Sciences, Zhejiang Chinese Medical University, Hangzhou, China

**Keywords:** ulcerative colitis, andrographolide, oxidative stress, inflammation, Nrf2/HO-1 pathway

## Abstract

Ulcerative colitis (UC) is a recurring inflammatory bowel disease, in which oxidative stress plays a role in its progression, and regulation of the oxidative/antioxidative balance has been suggested as a potential target for the treatment of UC. The aim of this study was to evaluate the protective effect of andrographolide against UC and its potential antioxidant properties by modulating the nuclear factor erythroid 2-related factor 2 (Nrf2)/heme oxygenase-1 (HO-1) pathway. Dextran sulfate sodium (DSS) -induced UC mice and the LPS-induced HT29 inflammatory cell model were established to uncover the potential mechanisms of andrographolide. ML385, a Nrf2 inhibitor, was used in both models to assess whether andrographolide exerts a protective effect against UC through the Nrf2/HO-1 pathway. The *in vivo* experiment showed that andrographolide ameliorated the symptoms and histopathology of DSS-induced mice and restored the expressions of ZO-1, Occludin-1 and Claudin-1. Meanwhile, DSS-induced oxidative stress and inflammation were suppressed by andrographolide treatment, along with the upregulation of key proteins in the Nrf2/HO-1 pathway. *In vitro* experiments showed that andrographolide attenuated LPS-induced excessive generation of ROS in HT29 cells, reduced inflammatory factors, and upregulated the expression of proteins related to tight junctions and Nrf2/HO-1 pathway. In addition, ML385 abolished the beneficial effect of andrographolide. In conclusion, the protective effect of andrographolide against UC may involve the suppression of oxidative stress and inflammation via the Nrf2/HO-1 pathway.

## 1 Introduction

Ulcerative colitis (UC) is a recurrent inflammatory condition in the gastrointestinal tract defined by symptoms like abdominal pain, diarrhea, inflammation of the mucosa, and bloody stools (Kaenkumchorn and Wahbeh, 2020). There has been an increase in the prevalence of UC in recent years, leading to a significant impact on quality of life for individuals due to its prolonged course and recurrent episodes (Le et al., 2023). Current pharmacological strategies for the treatment of UC focus on induction of remission and management of complications, including anti-inflammatory medications (such as mesalazine, sulfonamides), corticosteroids (like prednisolone, budesonide), immunosuppressants (such as azathioprine), and biologics (such as monoclonal antibodies) (Segal et al., 2021). Nevertheless, the extended utilization of these treatments is connected with diverse adverse reactions, such as nausea, vomiting, headache, diarrhoea, haematuria, nephrotoxicity, hypokalemia, increased susceptibility to infection and poor tolerability, (Seyedian et al., 2019; Kucharzik et al., 2020), limiting their clinical utility. Hence, it is imperative to investigate alternative therapeutic options that are both reliable and efficacious. Natural products with a long history of safe use in traditional medicine or diet can offer promising avenues of investigation (Ekiert and Szopa, 2020). Accordingly, the search for novel natural compounds and understanding their mechanisms of action in combating inflammation hold significant promise for the treatment of UC.

There is a common belief that the pathogenesis of UC is the result of a combination of factors such as genetic susceptibility, immune system dysfunction and intestinal flora dysbiosis (Du and Ha, 2020). The role of oxidative stress on the development of UC has received increasing attention (Jeon et al., 2020; Peng et al., 2023). Oxidative stress, characterized by an imbalance between oxidative and antioxidative, has been implicated in promoting inflammation by attracting neutrophils, increasing protease secretion, and generating high levels of oxidative byproducts (Forman and Zhang, 2021). Reactive oxygen species (ROS) are major reactive substances in the process of oxidative stress. It is a vital signaling molecule involved in mitogenic reactions or protection against infectious pathogens at low concentrations under normal conditions, but excessive ROS production can trigger oxidative stress, which in turn causes or exacerbates inflammation, DNA damage and even disease progression (Iantomasi et al., 2023; Jomova et al., 2023). During the pathogenesis of UC, inflammatory stimuli may lead to the overexpression of ROS, which can directly damage colonic tissue and exacerbate the symptoms of UC. Regulation of oxidative/antioxidative balance is expected to be one of the targets for effective treatment of UC (Pu et al., 2014; Liu et al., 2023).

Nuclear factor erythroid 2-related factor 2 (Nrf2), a member of the CNC family of transcription factors with a leucine zipper structure, is a key player in the antioxidant stress response (Wang et al., 2022). Translocating Nrf2 to the nuclear triggers the activation of antioxidant enzymes, improves ROS scavenging capacity and reduces oxidative stress damage (Keleku-Lukwete et al., 2018). HO-1 is a target gene of Nrf2 that induces ROS reduction. NF-KB has the ability to stimulate the production of inflammatory cytokines such as IL-10, IL-6, TNF-α, IL-8, *etc.*, thus mediating the inflammatory reaction (Poma, 2020). HO-1 can block NF- κB activation, leading to an indirect suppression of the inflammatory response (Zeng et al., 2023). Activated Nrf2 is the main mediator of cellular resistance to oxidative stress damage and can regulate antioxidant proteins, anti-degeneration proteins, drug transporter proteins, biotransformation enzymes, proteasomes and other cellular protective genes to protect cells from oxidative damage (Ma, 2013). The Nrf2 signaling pathway not only promotes the release of various antioxidant enzymes, but also inhibits the activation of pathways connected to inflammation and reduces the release of several inflammatory factors, which are crucial in managing UC (Bourgonje et al., 2023; Peng et al., 2023).

Andrographis paniculata (Burm. f.) Nees is a widely used medicinal herb in China and Southeast Asia (Jiang et al., 2021). It is a plant of the Acanthaceae family known for its ability to remove heat and toxins, cool the blood and reduce swelling (Kumar et al., 2021). Andrographolide, the primary diterpene lactone found in Andrographis paniculate (Patil and Jain, 2021), exhibits various biological effects, including anti-inflammatory (Burgos et al., 2020; Li et al., 2022), antiviral (Dhawan, 2012; Khanal et al., 2021), anti-apoptotic (Wanandi et al., 2020) and anti-tumor (Tundis et al., 2023). The anti-inflammatory properties of andrographolide against UC have been validated in both *in vitro* and *in vivo* experimental models. In our preliminary animal studies, andrographolide was found to inhibit the IL-23/IL-17 axis, reduce the disease activity index score of TNBS-induced colitis and promote intestinal mucosal repair (Zhu et al., 2018). However, the mechanism of andrographolide in UC has not been fully elucidated. This research aimed to investigate whether andrographolide can protect intestinal barrier integrity by attenuating oxidative stress and regulating the Nrf2 pathway.

## 2 Materials and methods

### 2.1 Animals and experimental design

C57BL/6J female mice (6 weeks old, 20 ± 2 g) were sourced from Shanghai Slac Laboratory Animal Co. Ltd. (Shanghai, China). The mice were kept under standard ambient conditions (temperature: 25°C ± 3°C, humidity: 53% ± 3%, and a 12 -hour of light/dark cycle) with unrestricted access to food and water for a week prior to the experiment to acclimate to the laboratory condition. The experimental protocols fully complied with the Guidelines for the Care and Use of Laboratory Animals (Chinese Council on Animal Research and the Guidelines of Animal Care) and approved by the Animal Welfare Committee of Zhejiang Chinese Medical University (certificate number IACUC-20220321-25).

Andrographolide (purity ≥98%, Sigma-Aldrich, United States) and ML385(Selleck Chemicals, United States) were dissolved in 0.5% CMC-Na. Dextran sulphate sodium (DSS, MV40000, Aladdin, Shanghai, China) was dissolved in distilled water.

The current study includes two different mouse experiments. To evaluate the effect of andrographolide on colitis, mice were divided into five groups (n = 10): normal control group (Control), DSS model group (DSS), and three groups receiving different doses of andrographolide treatment (DSS + Andro L, DSS + Andro M, and DSS + Andro H). Except for the control group, mice in the other groups drank water containing 3% (w/v) DSS consecutively for 14 days. The mice in the andrographolide treatment groups were orally administered with different doses of andrographolide (10, 20, and 40 mg/kg) respectively once daily from day 8 to day 14. To assess the role of Nrf2 in the effect of andrographolide on colitis, mice were divided into four groups (n = 10): normal control group (Control), DSS model group (DSS), andrographolide treatment group (DSS + Andro), and andrographolide and ML385 treatment group (DSS + Andro + ML385). Mice in the DSS, DSS + Andro and DSS + Andro + ML385 group were given water containing 3% (w/v) DSS for 14 days. From day 8 to day 14, mice in the DSS + Andro group were orally administered with 40 mg/kg andrographolide once daily. On day 8, 11, and 14, mice in the DSS + Andro + ML385 group were administered ML385 (30 mg/kg) by oral gavage. At the end of the experiment, colon tissues were collected from the mice and the colon length was measured.

### 2.2 Cell culture and treatment

HT29 cell lines were obtained from American Type Culture Collection (ATCC, Mannssas, VA) and cultured in RPMI-1640 supplemented with 10% fetal bovine serum (FBS) at 37°C in a cell incubator containing 5% (v/v) CO_2_. LPS was dissolved in the media and cells were primed for 2 h at a concentration of 8 μg/mL. After removing the priming media, cells were treated with different doses of andrographolide (10, 20, 40 μM) or ML385 (10 μM) for 8 h and collected for further experiments.

### 2.3 Histological analysis

The colon samples were extracted, fixed in 4% paraformaldehyde, dehydrated with gradient ethanol, and embedded in paraffin. Slices with a thickness of 5 μm were cut from the specimen and stained with hematoxylin and eosin (HE). The histology of the colon was observed under a microscope (ECLIPSE C1; Nikon Corporation, Tokyo, Japan).

### 2.4 Immunohistochemical staining

The above embedded samples were cut into 5 μm slices, deparaffinised, dehydrated and subjected to antigen recovery by immersion in citrate buffer at 98°C for 15 min to block endogenous peroxidase activity. Thereafter, the samples were treated with H_2_O_2_ and blocked with 3% bovine serum albumin (BSA) solution for 30 min. The samples were then hybridised with anti-myeloperoxidase (MPO; 1:200, ab208670, Abcam). After 12 h, the sections were incubated with the corresponding secondary antibody and then stained using a 3,3′-diaminobenzidine (DAB) staining kit (Vector Laboratories, United States).

### 2.5 Detection of serum LPS concentration

Levels of serum LPS was detected with an enzyme-linked immunosorbent assay kit (Cloud-Clone Corp., Wuhan, China) following the manufacturer’s directions.

### 2.6 Real-time PCR

RNA was extracted from colonic tissues or cells using TRIzol reagent (Invitrogen, CA) and RNA concentration was measured using Nanodrop (Thermo Scientific, MA). RNA was then converted to cDNA by a reverse transcription kit. Genes were amplified using the StepOnePlus real-time PCR system (Applied Biosystems, Foster City, CA, United States). Relative gene expressions were calculated using 2^− (△△CT)^ method. The primer sequences used in this study are shown in Table 1.

**TABLE 1:** Primer sequences for qPCR.

Gene	Description	Sequence (5′-3′)
mouse *IL-17*	Forward	TTT​AAC​TCC​CTT​GGC​GCA​AAA
	Reverse	CTT​TCC​CTC​CGC​ATT​GAC​AC
mouse *IL-23*	Forward	ATG​CTG​GAT​TGC​AGA​GCA​GTA
	Reverse	ACG​GGG​CAC​ATT​ATT​TTT​AGT​CT
mouse *IL-1*β	Forward	GCA​ACT​GTT​CCT​GAA​CTC​AAC​T
	Reverse	ATC​TTT​TGG​GGT​CCG​TCA​ACT
mouse *IL-6*	Forward	TAG​TCC​TTC​CTA​CCC​CAA​TTT​CC
	Reverse	TTG​GTC​CTT​AGC​CAC​TCC​TTC
mouse *TNFα*	Forward	CCC​TCA​CAC​TCA​GAT​CAT​CTT​CT
	Reverse	GCT​ACG​ACG​TGG​GCT​ACA​G
mouse *ZO-1*	Forward	GCC​GCT​AAG​AGC​ACA​GCA​A
	Reverse	TCC​CCA​CTC​TGA​AAA​TGA​GGA
mouse *Occludin-1*	Forward	TTG​AAA​GTC​CAC​CTC​CTT​ACA​GA
	Reverse	CCG​GAT​AAA​AAG​AGT​ACG​CTG​G
mouse *Claudin-1*	Forward	GGG​GAC​AAC​ATC​GTG​ACC​G
	Reverse	AGG​AGT​CGA​AGA​CTT​TGC​ACT
mouse *β-Actin*	Forward	GGC​TGT​ATT​CCC​CTC​CAT​CG
	Reverse	CCA​GTT​GGT​AAC​AAT​GCC​ATG​T

### 2.7 Western blot

Proteins from colon samples or cells were extracted using lysis buffer containing protease and phosphatase inhibitors. Protein concentrations were measured with the BCA assay kit (Beyotime, China). The proteins were then separated by SDS-PAGE, transferred to the PVDF membrane, and blocked with 5%BSA. Subsequently, primary antibodies were incubated. The antibodies were used as follows: anti-IL-17, anti-IL23, anti-ZO-1, anti-Occludin-1, and anti-Claudin-1 (Abcam, Cambridge, United Kingdom); anti-Nrf2, anti-HO-1, anti-IL-1β, anti-P-p65, anti-p65 (Cell Signaling Technology, Danvers, MA, United States); anti-β-actin (Boster Biological Technology, Pleasanton, CA, United States).β-actin was used as loading controls. Blots were developed using the appropriate HRP-conjugated secondary antibody and ECL kit (Nanjing Vazyme Biotech Co. Ltd., Nanjing, China) to detect.

### 2.8 Measurement of oxidative stress

To measure the antioxidant stress effect of andrographolide on colitis, colon tissues were homogenized in cold physiological saline and the levels of malonaldehyde (MDA), superoxide dismutase (SOD) and glutathione (GSH) in colon tissues were determined using commercial reagent kit (Nanjing Jiancheng Biotechnology Co., Ltd., Nanjing, China) according to the manufacturer’s instructions.

### 2.9 Detection of ROS

After treatment as described in 2.2, cells were collected and incubated in 5 μM DCFH-DA (Beyotime, Shanghai, China) at 37°C for 20 min, then washed by 3 times with serum-free cell culture medium to remove DCFH-DA that had not entered the cells. Finally, flow cytometry (BD Accuri C6, United States) and FlowJo software (version 10.0.7r2) were employed to evaluate the ROS levels.

### 2.10 Statistical analysis

Mean values ± SEM were used to express the data. GraphPad Prism 8 software (San Diego, CA, United States) was employed for statistical analysis. Normality test was performed using Shapiro-Wilk test. If data passed this test, we used unpaired Student t test followed by Welch’s corrections. For more than two groups, one-way analysis of variance (ANOVA) with a *post hoc* Turkey’s test for multiple comparisons was performed. Statistical significance was considered at *p* < 0.05.

## 3 Results

### 3.1 Andrographolide protected mice from colitis induced by DSS

**FIGURE 1:**
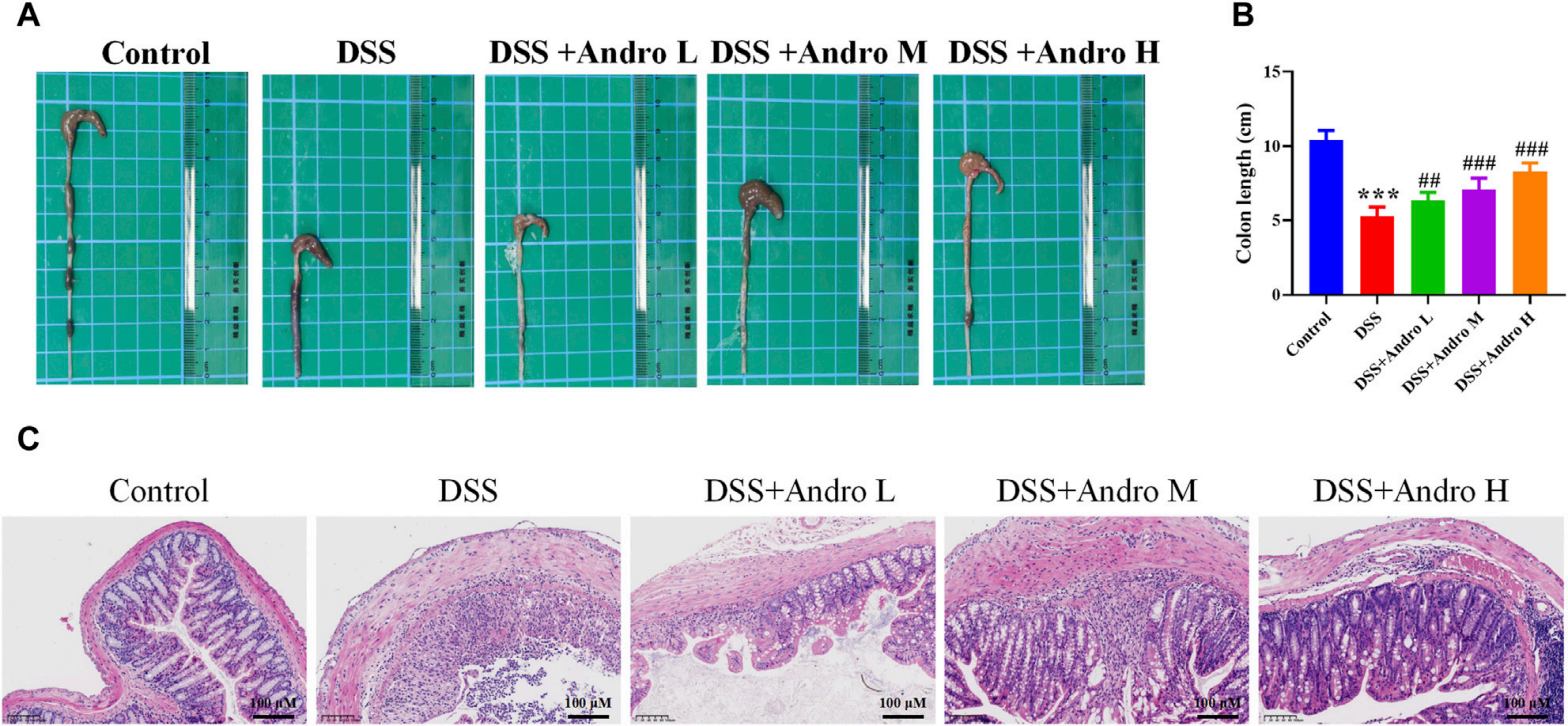
Andrographolide ameliorated DSS-induced colitis in mice. **(A)** Representative images of the colon. **(B)** Colon length. **(C)** Pictures of H&E staining (amplification 200, bar = 100 μM). The data are presented as mean ± SD (n = 6). ***p < 0.001, compared with control group; ##*P* < 0.01, ###*P* < 0.001, compared with the DSS group.

To investigate the therapeutic effect of andrographolide on DSS-induced colitis, colon length was measured. As shown in Figures 1A,B, the colon length in the DSS group was shorter than that in the control group (*P* < 0.001). After treatment with different doses of andrographolide, the colon length was significantly increased (*P* < 0.01, *P* < 0.001). The pathological result showed that the colon tissue of the control group presented a clear and intact structure. However, the DSS-induced colitis group showed a disordered structure with infiltration of inflammatory cells or crypt damage, loss of goblet cells. Andrographolide could effectively alleviate these pathological changes and high dose andrographolide administration showed minimal histological damage (Figure 1C).

### 3.2 Andrographolide increased tight junction proteins *in vivo* and *in vitro*

**FIGURE 2:**
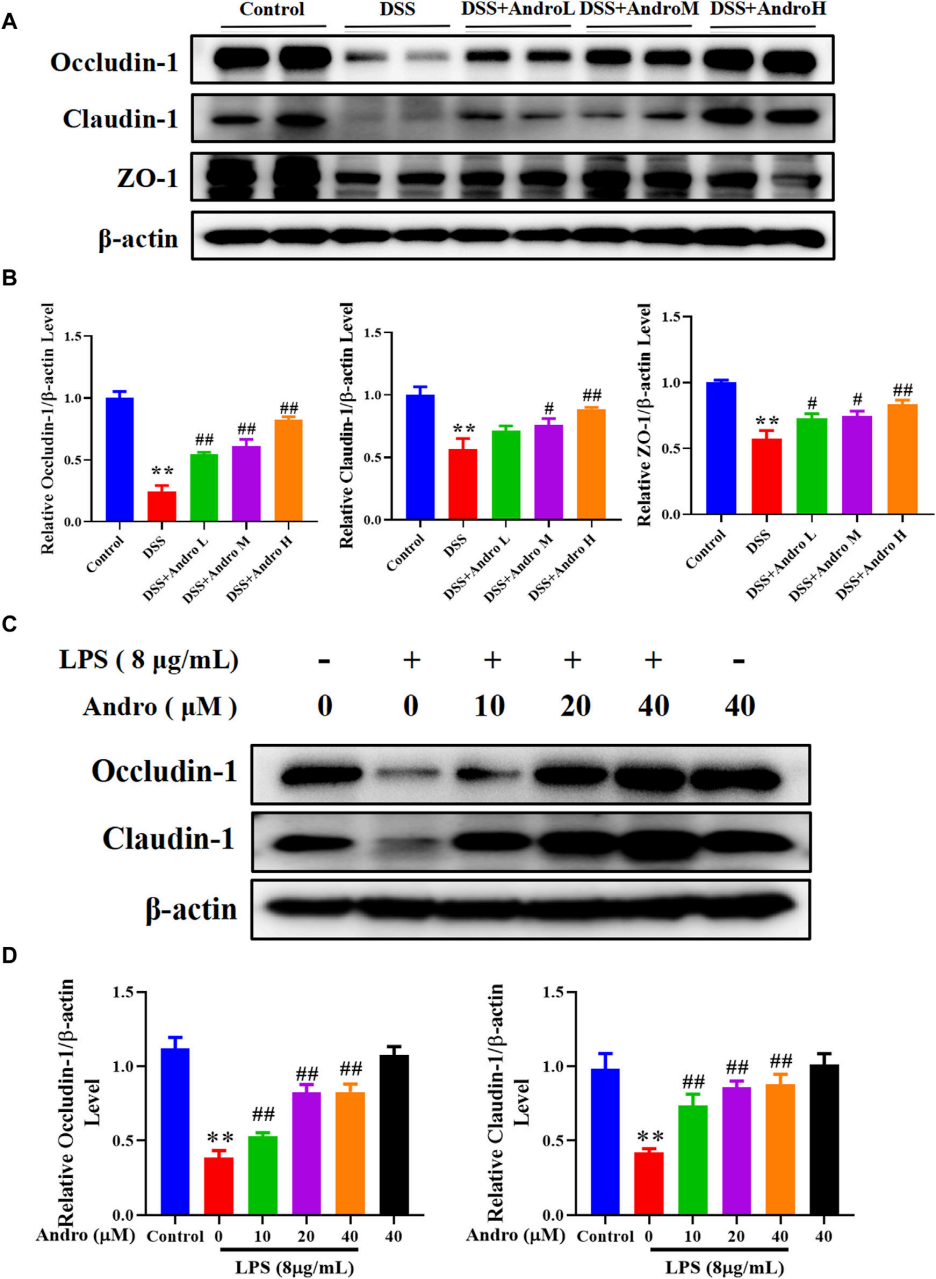
Andrographolide exerted protective effect on intestinal barrier of UC. **(A)** The protein expressions of Occludin-1, Claudin-1, and ZO-1 in mice colon tissues. **(B)** Quantitation of the expression of Occludin-1, Claudin-1, and ZO-1 protein. The results are presented as mean ± SD (n = 3). ^*^ *P* < 0.05, ^**^ *P* < 0.01, compared with control group; ^#^ *P* < 0.05, ^##^ *P* < 0.01, compared with the DSS group. **(C)** The protein expressions of Occludin-1 and Claudin-1 in HT29 cells. **(D)** Quantitation of the expression of Occludin-1 and Claudin-1 protein. The results are presented as mean ± SD (n = 3). ^**^ *P* < 0.01, compared with control group; ^#^ *P*< 0.05, ^##^ *P* < 0.01, compared with the LPS group.

Tight junction proteins are essential for intestinal barrier integrity in colitis. As shown in Figures 2A,B, DSS induction damaged the intestinal barrier and decreased the protein expressions of Occludin-1, Claudin-1 and ZO-1 (*P* < 0.01). After treatment with andrographolide at different doses (10, 20, and 40 mg/kg), the expressions of Occludin-1 and ZO-1 increased significantly in a concentration-dependent manner compared with the DSS group (*P* < 0.05, *P* < 0.01).Treatment with andrographolide at the concentration of 20 mg/kg and 40 mg/kg showed a significant increase in the levels of Claudin-1 compared with the DSS group (*P* < 0.05, *P* < 0.01). However, no effect was documented when andrographolide was tested at doses of 10 mg/kg. In addition, the *in vitro* experiment also showed that andrographolide treatment at concentrations of 10, 20, and 40 μM counteracted the barrier impairment induced by LPS in HT-29 cells, showing an increased protein expressions of Occludin-1 and Claudin-1 (*p* < 0.01, Figures 2C,D).

### 3.3 Andrographolide suppressed inflammatory factors both *in vivo* and *in vitro*

**FIGURE 3:**
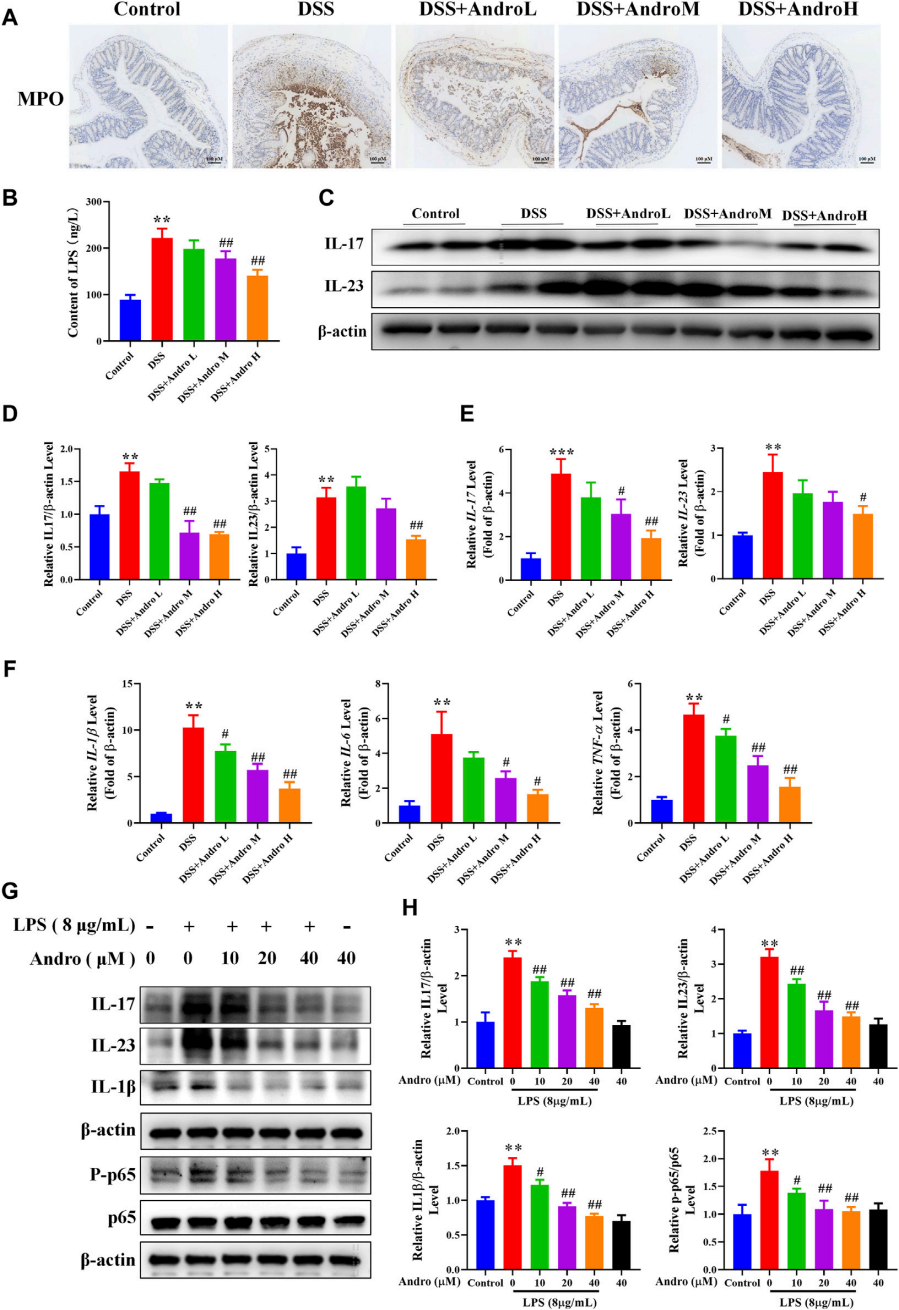
Andrographolide modulated the levels of inflammatory cytokines. **(A)** The expression of MPO in mice colon tissues (amplification 100, bar = 100 μM). **(B)** The levels of serum LPS. **(C)** The protein expressions of IL-17 and IL-23 in mice colon tissues. **(D)** Relative levels of IL-17 and IL-23 expressions. **(E)** The mRNA expressions of IL-17 and IL-23 in mice colon tissues. **(F)** The mRNA expressions of IL-1β, IL-6 and TNFα. The results are presented as mean ± SD (n = 3–5). ^*^ *P* < 0.05, ^**^ *P* < 0.01, ^***^ *p* < 0.001, compared with control group; ^#^ *P* < 0.05, ^##^ *P* < 0.01, compared with the DSS group. **(G)** The protein expressions of IL-17, IL-23, IL-1β and p-p65 in LPS primed HT-29 cells. **(H)** Relative levels of IL-17, IL-23, IL-1β and p-p65. The results are presented as mean ± SD (n = 3). ^**^ *P* < 0.01, compared with control group; ^#^ *P* < 0.05, ^##^ *P* < 0.01, compared with the LPS group.

MPO, the main component of neutrophils, is a heme protein synthesized during the differentiation process of granulocytes. To detect neutrophil infiltration into the intestine, we detected the expression of MPO in colon tissue. The result of Figure 3A showed that there were more yellow stained particles in the intestinal mucosa of DSS group mice compared to the normal group, indicating an increase in neutrophil infiltration after DSS treatment. As the treatment with different doses of andrographolide, the number of yellow stained particles gradually decreased, suggesting that andrographolide can reduce neutrophil infiltration.

LPS is a large molecular bacterial endotoxin that can serve as an indicator of intestinal permeability. Due to the destruction of the intestinal mucosa in colitis mice, the large molecule bacterial product LPS can enter the bloodstream, leading to long-term stimulation of the immune system and intestinal inflammation. In this study, after DSS treatment, the levels of serum LPS significantly increased (*P* < 0.01). While andrographolide at the dose of 20 mg/kg and 40 mg/kg significantly reduced serum LPS levels (*P* < 0.01) (Figure 3B).

We also determined the effect of andrographolide on the IL-23/IL-17 axis. As shown in Figures 3C–E, the protein and mRNA expressions of IL-23 and IL-17 were higher in mice of the DSS group than those of the control group (*P* < 0.001, *P* < 0.01, *P* < 0.05). Nevertheless, the expression of IL-17 was notably reduced by andrographolide (20 and 40 mg/kg) (*P* < 0.05, *P* < 0.01) and the expression of IL-23 was decreased in mice treated with high dose of andrographolide (*P* < 0.01, *P* < 0.05). The mRNA levels of the pro-inflammatory cytokines IL-1β, IL-6 and TNF-α were significantly higher in the DSS group than in the control group (*P* < 0.01). Compared with the DSS group, andrographolide treatment at different doses conspicuously attenuated the levels of IL-1β, IL-6, and TNF-α (*P* < 0.05, *P* < 0.01) (Figure 3F). In addition, as shown in Figures 3G,H, the *in vitro* experiment showed that the increased protein expressions of IL-17, IL-23, IL-1β, and p-p65 in LPS-primed HT-29 cells were sharply reduced by andrographolide at different doses (*P* < 0.05, *P* < 0.01). These data suggest that andrographolide plays a vital anti-inflammatory role in colitis.

### 3.4 Andrographolide inhibited oxidative stress *in vivo* and *in vitro*

**FIGURE 4:**
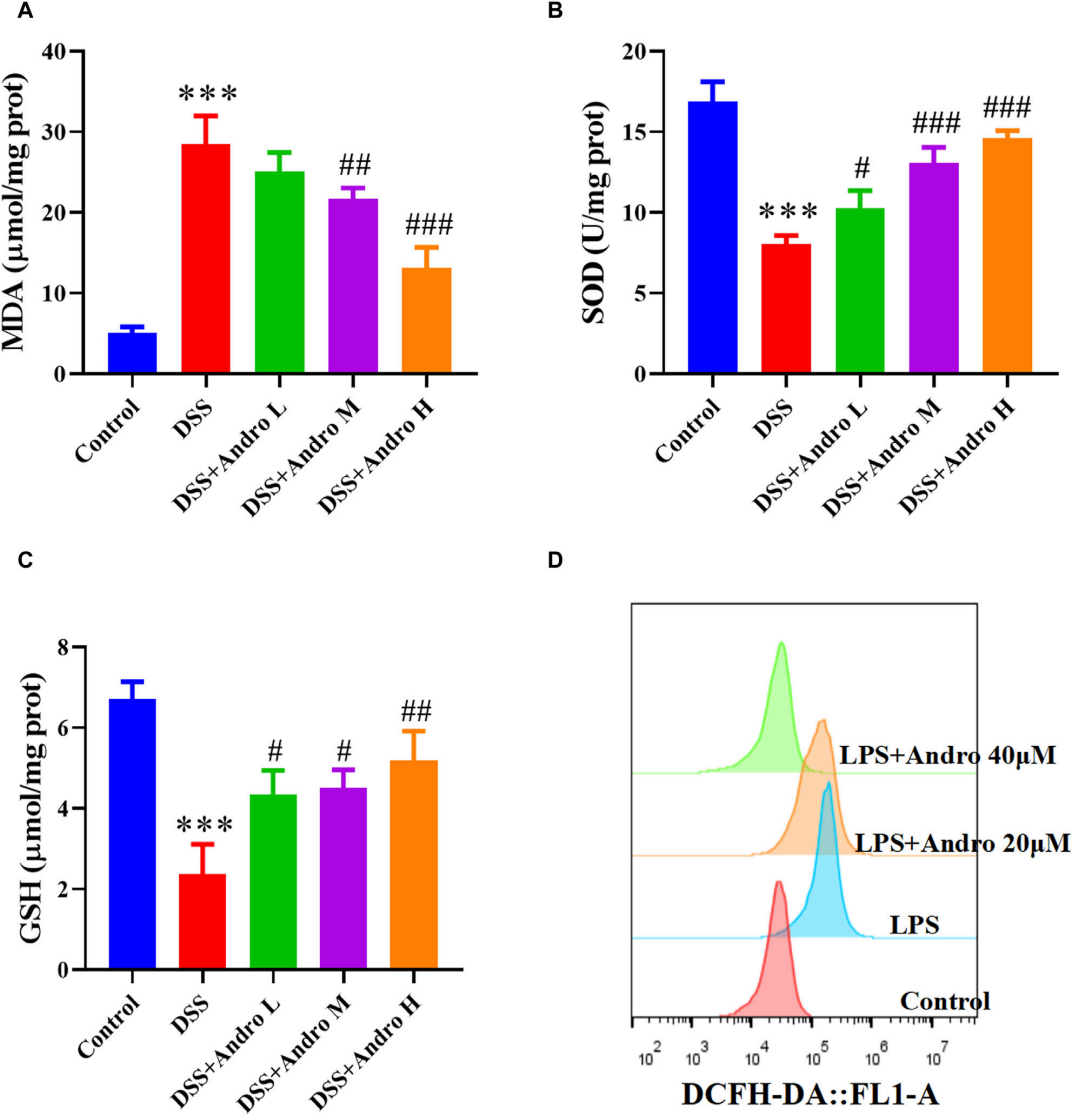
Andrographolide inhibited oxidative stress. Effect of andrographolide on the content of MDA **(A)**, the activity of GSH **(B)** and SOD **(C)** level in the colonic tissues. The data are presented as mean ± SD (n = 5). ^***^ *P* < 0.001, compared with control group; ^#^ *P* < 0.05, ^##^ *P* < 0.01, ^###^ *P* < 0.001, compared with the DSS group. **(D)** Representative images of ROS level in HT29 cells.

Oxidative stress is a crucial factor in the progression of colitis. In order to examine the impact of andrographolide on oxidative stress, we analyzed the levels of MDA, as well as antioxidant enzymes such as SOD and GSH. As shown in Figures 4A–C, the level of MDA was elevated in colonic tissues of DSS-induced mice, whereas the levels of SOD and GSH were strikingly reduced compared to the control group (*P* < 0.001). Administration of andrographolide increased MDA level at doses of 20 and 40 mg/kg and decreased SOD and GSH levels at doses of 10, 20, and 40 mg/kg. In addition, DCFH-DA prob indicated that LPS elevated the level of ROS in HT-29 cells. However, treatment with andrographolide remarkably reversed these effects (Figure 4D).

### 3.5 Andrographolide activated the Nrf2/HO-1 signaling pathway *in vivo* and *in vitro*

**FIGURE 5:**
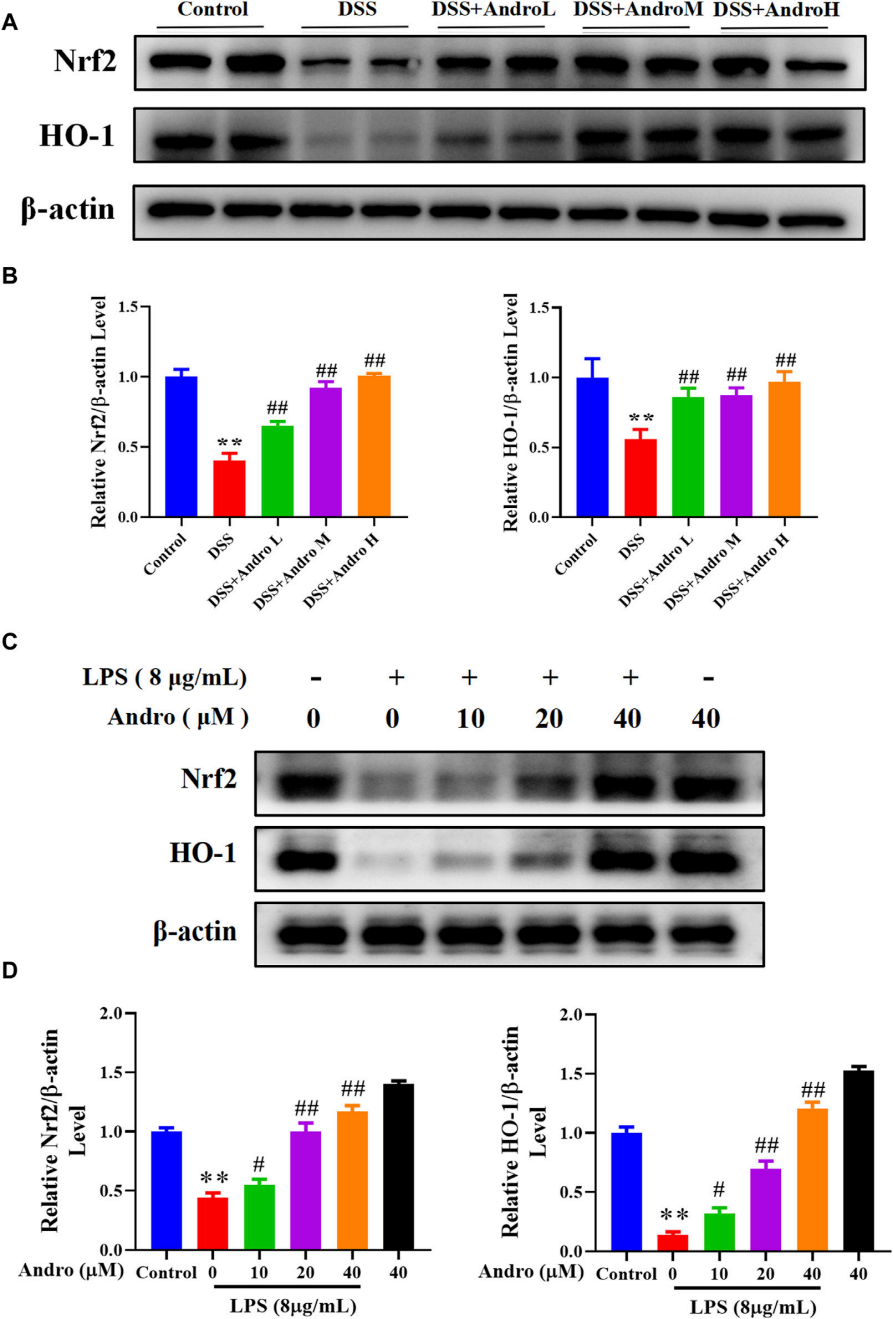
Andrographolide activated the Nrf2/HO-1 signaling pathway. **(A)** The protein expressions of Nrf2 and HO-1 in mice colon tissues. **(B)** Relative levels of Nrf2 and HO-1 expressions. The results are presented as mean ± SD (n = 3). ^**^ *P* < 0.01, compared with control group; ^##^ *P* < 0.01, compared with the DSS group. **(C)** The protein expressions of Nrf2 and HO-1 in LPS primed HT-29 cells. **(D)** Relative levels of Nrf2 and HO-1 expressions. The results are presented as mean ± SD (n = 3). ^**^ *P* < 0.01, compared with control group; ^#^ *P* < 0.05, ^##^ *P* < 0.01, compared with the LPS group.

To investigate the regulation of andrographolide on the Nrf2/HO-1 signaling pathway, the Western blot experiment was performed. The result showed that DSS exposure decreased the protein expressions of Nrf2 and HO-1 (*P <* 0.01). In contrast, treatment with different doses of andrographolide observably increased the expressions of Nrf2 and HO-1 compared with the DSS group *in vivo* (*P* < 0.01, Figures 5A,B). The *in vitro* experiment also demonstrated that HT-29 cells treated with LPS exhibited significantly reduced levels of Nrf2 and HO-1 (*P <* 0.01), while different concentrations of andrographolide (10, 20, 40 μM) treatment upregulated the expressions of Nrf2 and HO-1 (*P* < 0.05, *P <* 0.01, Figures 5C,D).

### 3.6 Inhibition of Nrf2 abolished andrographolide-mediated improvement *in vivo* and *in vitro*

**FIGURE 6:**
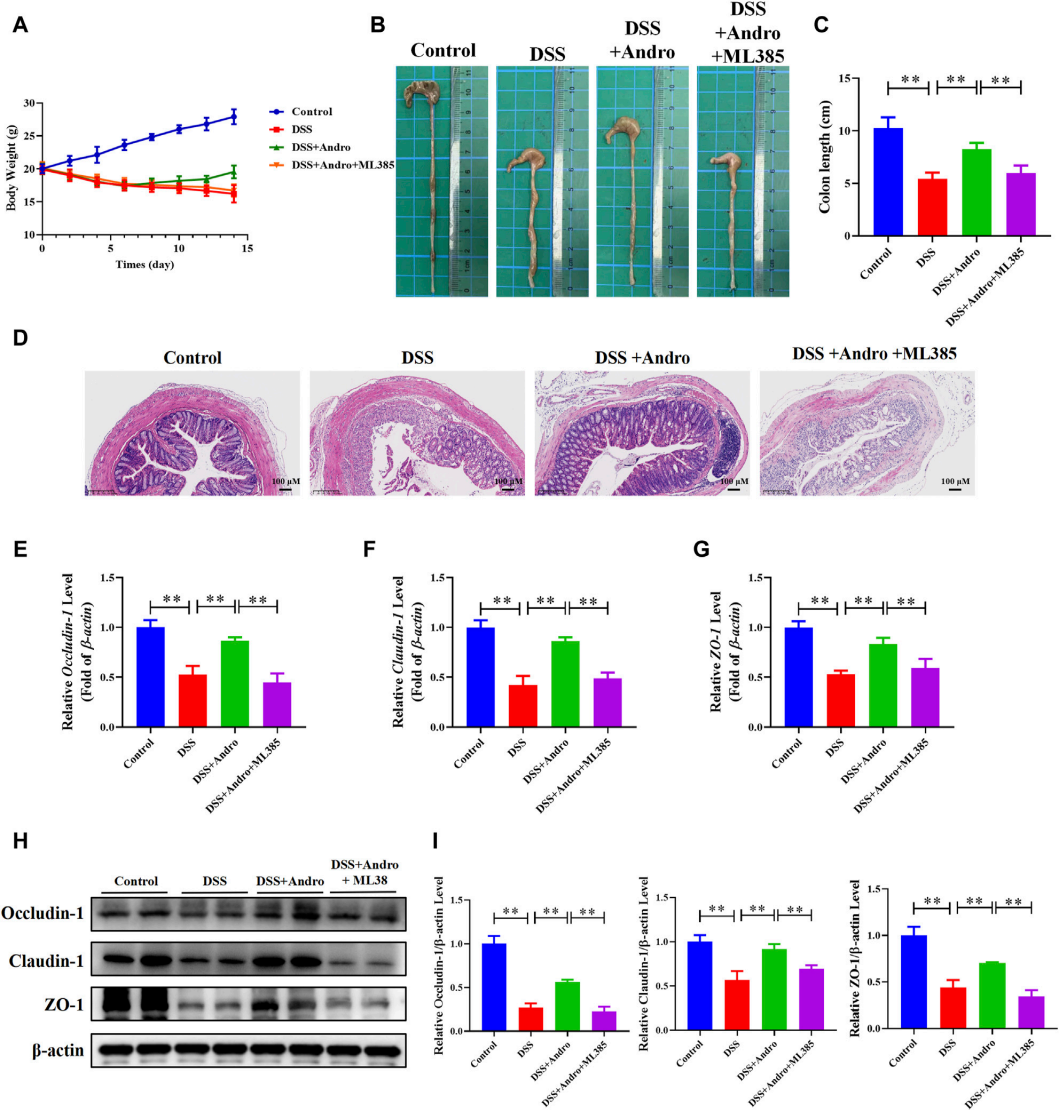
Inhibition of Nrf2 abolished the protective effects of andrographolide in DSS-induced colitis mice. **(A)** body weight. **(B)** Representative images of the colon. **(C)** Colon length. **(D)** Pictures of H&E staining (amplification 100, bar = 100 μM). **(E–G)** The mRNA levels of Occludin-1, Claudin-1, and ZO-1 in mice colon tissues. **(H)** The protein expressions of Occludin-1, Claudin-1, and ZO-1 in mice colon tissues. **(I)** Quantitation of the expression of Occludin-1, Claudin-1, and ZO-1 protein. The data are presented as mean ± SD (n = 3–10). ^*^ *P* < 0.05, ^**^ *P* < 0.01.

**FIGURE 7:**
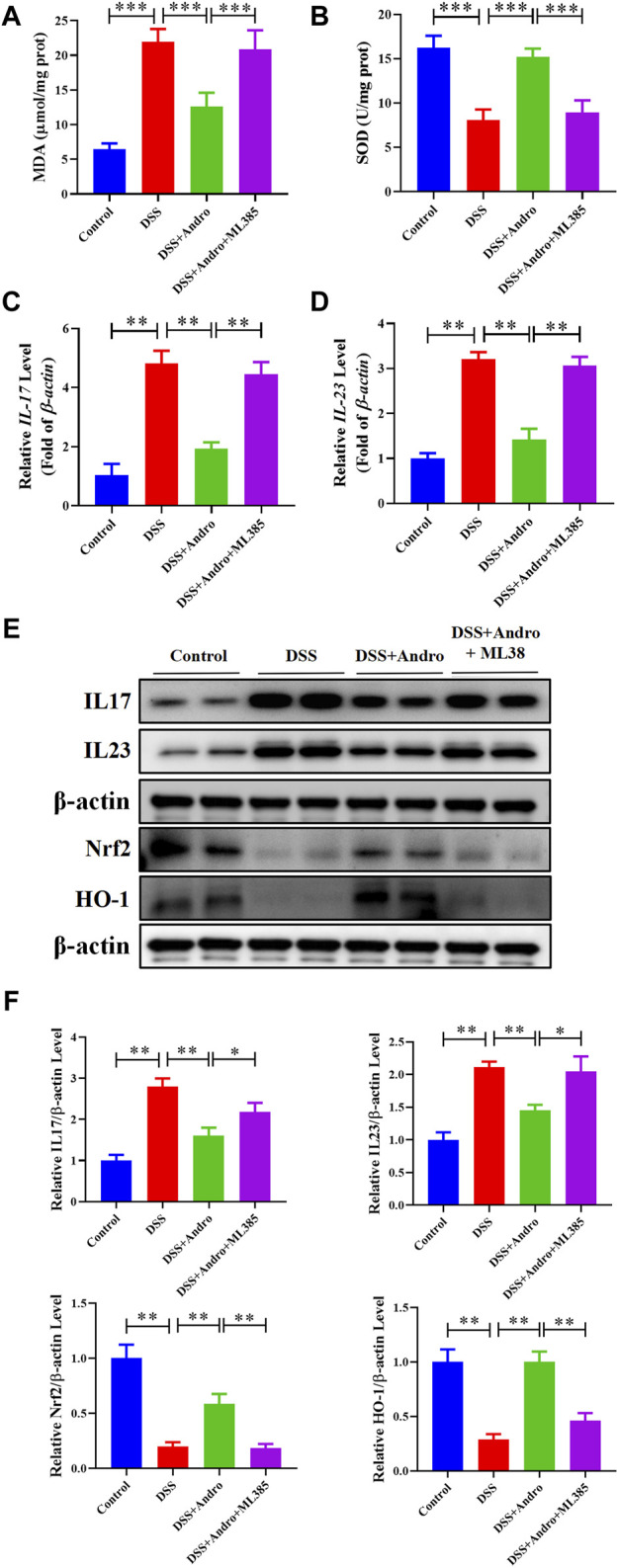
Inhibition of Nrf2 attenuated the protective effects of andrographolide on inflammatory factors and oxidative stress through Nrf2/HO-1 pathway in DSS-induced colitis mice. **(A, B)** The level of MDA and SOD. **(C, D)** The mRNA levels of IL-17 and IL-23 in mice colon tissues. **(E)** The protein expressions of IL-17, IL-23, Nrf2 and HO-1 in mice colon tissues. **(F)** Quantitation of the expression of IL-17, IL-23, Nrf2, and HO-1 protein. The data are presented as mean ± SD (n = 3–5). ^*^ *P* < 0.05, ^**^ *P* < 0.01.

**FIGURE 8:**
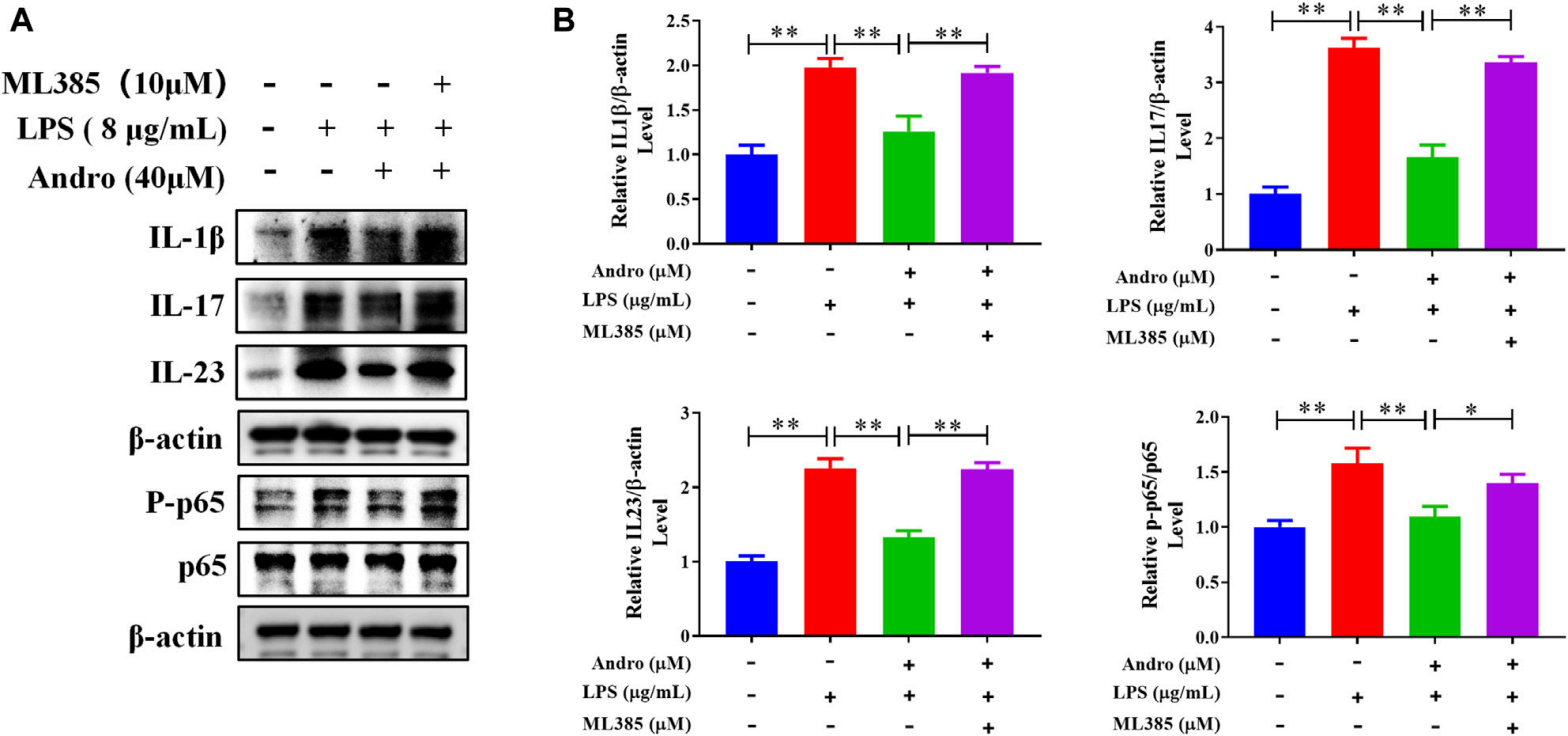
Inhibition of Nrf2 attenuated the protective effects of andrographolide on inflammatory factors. **(A)** The protein expressions of IL-1β, IL-17, IL-23, and P-p65 in HT29 cells. **(B)** Quantitation of the expression of IL-1β, IL-17, IL-23, and P-p65 protein. The data are presented as mean ± SD (n = 3). ^*^ *P* < 0.05, ^**^ *P* < 0.01.

To further confirm whether andrographolide protects against colitis through the Nrf2/HO-1 pathway, ML385, an inhibitor of Nrf2, was applied to co-treat *in vivo* and *in vitro*. As demonstrated in Figures 6A–I, compared with the andrographolide group, ML385 co-treatment could antagonize the effect of andrographolide on DSS-induced colitis including body weight, colon length, pathological changes and tight junction proteins of the colon. Meanwhile, co-treatment with ML385 attenuated the protective effects of andrographolide on inflammatory factors IL-17 and IL-23 and oxidative stress (Figures 7A–F). The results confirmed that Nrf2 levels in DSS-induced colitis were reduced after co-treatment with ML385, and subsequently the upregulated effect of andrographolide on HO-1 was also abolished by ML385 (Figures 7E,F). *In vitro* experiments also showed that ML385 eliminated inflammatory factors and NF-κB reduced by andrographolide treatment in HT29 cells treated with LPS (Figures 8A,B). Overall, these results indicated that andrographolide suppressed inflammatory factors and attenuated oxidative stress through the Nrf2/HO-1 signaling pathway.

## 4 Discussion

As an intractable disease in modern clinics, UC represents a grievous threat to human health (Gros and Kaplan, 2023). Inflammatory response of intestinal mucosa is a significant pathologic feature of UC. Researches show that IL-23/IL-17 axis may play a crucial role in the pathogenesis of UC (Dragasevic et al., 2018). During intestinal inflammation, intestinal bacterial antigens stimulate the antigen-presenting cells to secrete pro-inflammatory cytokines such as IL-23. Under its influence, CD4^+^ T cells are activated through the STAT3 pathway to control the differentiation of helper T cells into Th17 cells, and induce the expression of IL-17 gene and secretion of IL-17. IL-17 has a powerful ability to recruit and activate neutrophils, and stimulate fibroblasts, endothelial cells and macrophages to secrete various pro-inflammatory cytokines such as TNF-α, IL-1β and IL-6. In a normal intestinal environment, the primary role of Th17 cells may be protective, but in the pathogenesis of UC, highly expressed IL-23 activates the pathogenicity of IL-17, causing severe tissue damage in the intestine (Bunte and Beikler, 2019). Oxidative stress is also an important factor associated with the UC progression (Roessner et al., 2008). Oxidative stress refers to the process by which excessive free radicals or other reactive oxygen species present in the intracellular and extracellular environments leading to damage to cell structure and function (Schieber and Chandel, 2014). Free radicals are highly reactive ions or molecules with unpaired electrons that can engage in electron transfer reactions with other ions or molecules, leading to negative effects like lipid peroxidation in cell membranes and breakage of DNA (Gebicki, 2016). There is a close relationship between oxidative stress and inflammatory response. On the one hand, oxidative stress can induce inflammatory response, such as free radicals that activate macrophages and T lymphocytes, promoting the onset of inflammatory response. On the other hand, inflammatory reactions can also cause oxidative stress, since signaling molecules such as interleukin and tumor necrosis factor can stimulate NADPH oxidase, generating large number of free radicals (Srivastava et al., 2011; Lauridsen, 2019). In the inflamed region of the colon, elevated levels of inflammatory mediators and decreased anti-inflammatory factors result in the production and release of excessive ROS. This process also diminishes the effectiveness of free radical scavenging enzymes like SOD, ultimately leading to a continuous accumulation of ROS. Oxidative stress causes damage to the colonic mucosal barrier, which is a potential pathogenic factor in UC (Pereira et al., 2015). This study found that andrographolide reduced the levels of IL-23 and IL-17, further suppressing the neutrophil infiltration and secretion of inflammatory factors TNF-α, IL-1β, and IL-6. Meanwhile, andrographolide can significantly lower MDA, increase SOD and GSH activity, reduce intestinal mucosal oxidative stress, further inhibit inflammatory response, alleviate the symptoms of model mice, improve intestinal mucosal pathology, upregulate the levels of Claudin-1, ZO-1, and Occludin-1, and restore intestinal barrier function. This result indicates that andrographolide has a protective effect on intestinal mucosal barrier, which may be related to the inhibition of inflammatory response and oxidative stress.

The signaling pathway known as Nrf2/HO-1 is the major defense against oxidative damage (Loboda et al., 2016). Nrf2 serves as a vital regulator of cellular redox balance and is a pivotal player in responding to oxidative stress (Kensler et al., 2007). It can respond to exogenous damage and pathogenic molecules. During the physiological state, Nrf2 is primarily located in the cytoplasm and interacts with Keap1, which is in a suppressed condition. Upon activation by signals of oxidative stress, it quickly dissociates from Keap1 and translocates to the nucleus in a stable form to initiate antioxidant response elements (ARE) (Kang et al., 2004; Kobayashi and Yamamoto, 2006). Upon binding to ARE, transcription and translation of numerous genes downstream are initiated, upregulating the expression of antioxidants and alleviating oxidative stress responses caused by variety of damage agents (Baird and Dinkova-Kostova, 2011). HO-1, an enzyme that limits rates for heme breakdown, is found in various tissues and provides cell protection. More importantly, it is essential in responding to stress conditions (Cheng et al., 2015). As an inducible protein, it can increase exponentially when the body is in a stressful environment of ischemia and hypoxia. HO-1 and its metabolites have been reported to have significant antioxidant effects (Mahmoud et al., 2018). The main antioxidant effect of HO-1 is attributed to two key factors: firstly, HO-1 inhibits the involvement of free heme from in oxidative processes, and secondly, it collaborates with its breakdown byproducts, CO and bilirubin, to enhance its antioxidant and anti-inflammatory properties (Kongpetch et al., 2012). HO-1 is predominantly found in epithelial cells of the intestinal mucosa, where it serves to safeguard the intestinal mucosa by decreasing the infiltration of inflammatory cell (Paul et al., 2005). The pathway Nrf2/HO-1 is a vital antioxidant stress response pathway that plays a significant role in different areas, including the response to inflammation and the restoration of mucosal tissue (Zheng et al., 2023). Knockout of Nrf2 has been shown to exacerbate colon injury in mice. Nrf2^−/−^ mice were highly susceptible to DSS-induced UC because it could reduce antioxidant enzymes such as HO-1 and increase inflammatory factors like IL-6, TNF-α, and activation of Nrf2 could exert a protective effect on the colon (Khor et al., 2006; Osburn et al., 2007). The study demonstrated a significant decrease in the levels of Nrf2 and HO-1 proteins and mRNA in the colon tissue of colitis mice. After intervention with andrographolide, there was a noticeable increase in the expression of Nrf2 and HO-1. Following the administration of ML385, the impact of andrographolide was diminished, suggesting a close correlation between the Nrf2 signaling pathway and the pathogenesis of UC. To conclude, the suppressive effect of andrographolide on oxidative stress and inflammatory response in UC may be linked to the stimulation of the Nrf2/HO-1 signaling pathway and the restoration of the balance of the oxidative stress system.

## 5 Conclusion

**FIGURE 9:**
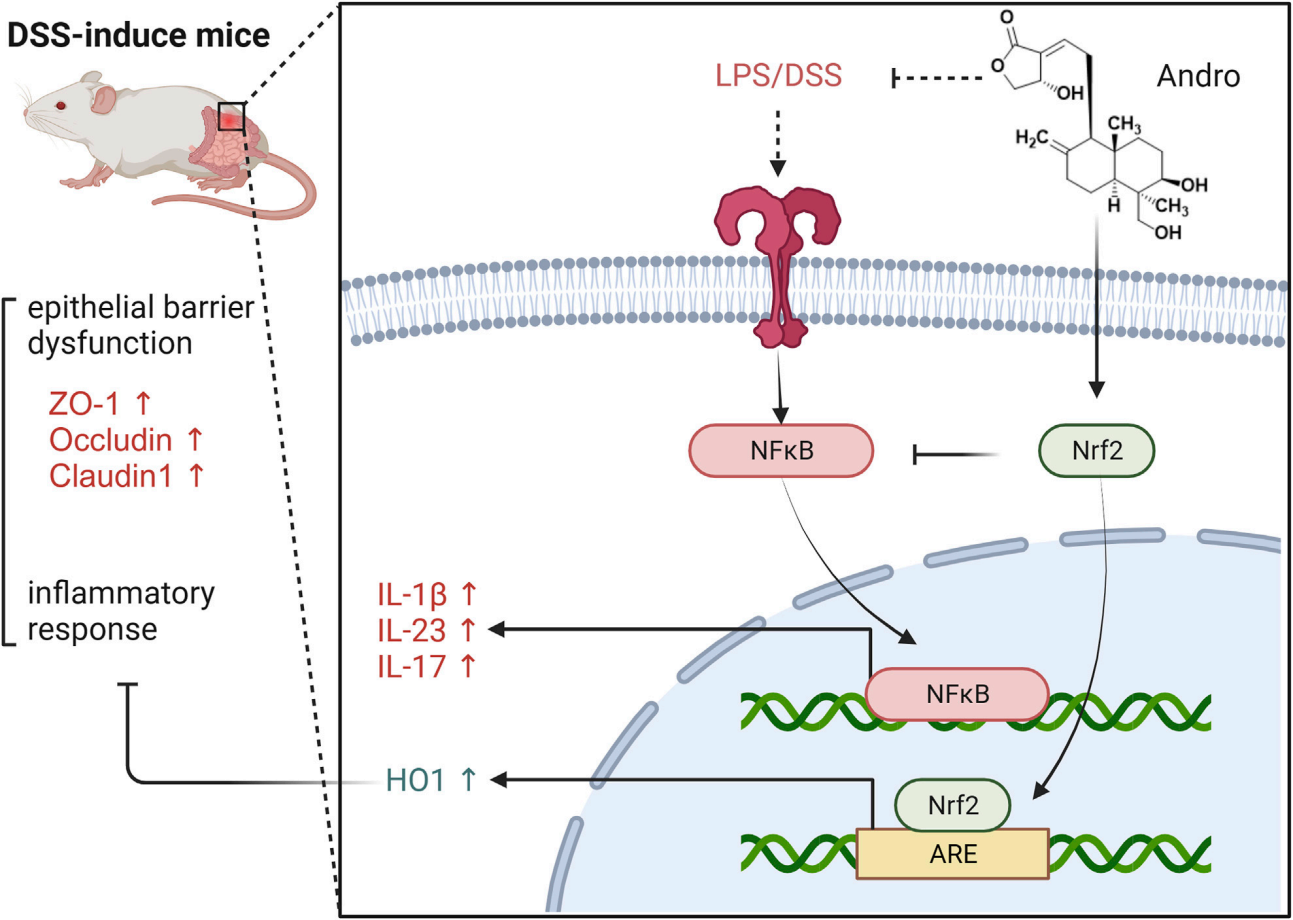
The protective effect of Andrographolide against ulcerative colitis by suppressing oxidative stress and inflammation through activation of the Nrf2/HO-1 pathway.

In summary, andrographolide can alleviate symptoms such as diarrhea and bloody stools in DSS induced colitis, repair damaged intestinal mucosal barriers, inhibit inflammatory factors and counteract oxidative stress responses. Moreover, andrographolide treats UC by activating the Nrf2/HO-1 signaling pathway. It was also discovered that the Nrf2 inhibitor ML385 eliminated the improvement effect of andrographolide on UC, indicating that the efficacy of andrographolide on reducing oxidative stress and improving UC symptoms is mainly through the Nrf2/HO-1 pathway (Figure 9).

## Data Availability

The raw data supporting the conclusions of this article will be made available by the authors, without undue reservation.

## References

[B1] Baird L. Dinkova-Kostova A. T. (2011). The cytoprotective role of the Keap1-Nrf2 pathway. Arch. Toxicol. 85, 241–272. 10.1007/s00204-011-0674-5 21365312

[B2] Bourgonje A. R. Kloska D. Grochot-Przęczek A. Feelisch M. Cuadrado A. van Goor H. (2023). Personalized redox medicine in inflammatory bowel diseases: an emerging role for HIF-1α and NRF2 as therapeutic targets. Redox Biol. 60, 102603. 10.1016/j.redox.2023.102603 36634466 PMC9841059

[B3] Bunte K. Beikler T. (2019). Th17 cells and the IL-23/IL-17 Axis in the pathogenesis of periodontitis and immune-mediated inflammatory diseases. Int. J. Mol. Sci. 20 (14), 3394. 10.3390/ijms20143394 31295952 PMC6679067

[B4] Burgos R. A. Alarcón P. Quiroga J. Manosalva C. Hancke J. (2020). Andrographolide, an anti-inflammatory multitarget drug: all roads lead to cellular metabolism. Molecules 26 (1), 5. 10.3390/molecules26010005 33374961 PMC7792620

[B5] Cheng H. T. Yen C. J. Chang C. C. Huang K. T. Chen K. H. Zhang R. Y. (2015). Ferritin heavy chain mediates the protective effect of heme oxygenase-1 against oxidative stress. Biochim. Biophys. Acta 1850, 2506–2517. 10.1016/j.bbagen.2015.09.018 26423448

[B6] Dhawan B. N. (2012). Anti-Viral activity of Indian plants. Proc. Natl. Acad. Sci. India Sect. B Biol. Sci. 82 (1), 209–224. 10.1007/s40011-011-0016-7 PMC7099914 32226204

[B7] Dragasevic S. Stankovic B. Sokic-Milutinovic A. Milosavljevic T. Milovanovic T. Lukic S. (2018). Importance of TLR9-IL23-IL17 axis in inflammatory bowel disease development: gene expression profiling study. Clin. Immunol. 197, 86–95. 10.1016/j.clim.2018.09.001 30193869

[B8] Du L. Ha C. (2020). Epidemiology and pathogenesis of ulcerative colitis. Gastroenterol. Clin. North Am. 49 (4), 643–654. 10.1016/j.gtc.2020.07.005 33121686

[B9] Ekiert H. M. Szopa A. (2020). Biological activities of natural products. Molecules 25 (23), 5769. 10.3390/molecules25235769 33297511 PMC7730830

[B10] Forman H. J. Zhang H. (2021). Targeting oxidative stress in disease: promise and limitations of antioxidant therapy. Nat. Rev. Drug Discov. 20 (9), 689–709. 10.1038/s41573-021-00233-1 34194012 PMC8243062

[B11] Gebicki J. M. (2016). Oxidative stress, free radicals and protein peroxides. Arch. Biochem. Biophys. 595, 33–39. 10.1016/j.abb.2015.10.021 27095212

[B12] Gros B. Kaplan G. G. (2023). Ulcerative colitis in adults: a review. JAMA 330 (10), 951–965. 10.1001/jama.2023.15389 37698559

[B13] Iantomasi T. Romagnoli C. Palmini G. Donati S. Falsetti I. Miglietta F. (2023). Oxidative stress and inflammation in osteoporosis: molecular mechanisms involved and the relationship with microRNAs. Int. J. Mol. Sci. 24 (4), 3772. 10.3390/ijms24043772 36835184 PMC9963528

[B14] Jeon Y. D. Lee J. H. Lee Y. M. Kim D. K. (2020). Puerarin inhibits inflammation and oxidative stress in dextran sulfate sodium-induced colitis mice model. Biomed. Pharmacother. 124, 109847. 10.1016/j.biopha.2020.109847 31981944

[B15] Jiang M. Sheng F. Zhang Z. Ma X. Gao T. Fu C. (2021). Andrographis paniculata (Burm.f.) Nees and its major constituent andrographolide as potential antiviral agents. J. Ethnopharmacol. 272, 113954. 10.1016/j.jep.2021.113954 33610706

[B16] Jomova K. Raptova R. Alomar S. Y. Alwasel S. H. Nepovimova E. Kuca K. (2023). Reactive oxygen species, toxicity, oxidative stress, and antioxidants: chronic diseases and aging. Arch. Toxicol. 97 (10), 2499–2574. 10.1007/s00204-023-03562-9 37597078 PMC10475008

[B17] Kaenkumchorn T. Wahbeh G. (2020). Ulcerative colitis: making the diagnosis. Gastroenterol. Clin. North Am. 49 (4), 655–669. Epub 2020 Sep 23. 10.1016/j.gtc.2020.07.001 33121687

[B18] Kang M. I. Kobayashi A. Wakabayashi N. Kim S. G. Yamamoto M. (2004). Scaffolding of Keap1 to the actin cytoskeleton controls the function of Nrf2 as key regulator of cytoprotective phase 2 genes. Proc. Natl. Acad. Sci. U. S. A. 101, 2046–2051. 10.1073/pnas.0308347100 14764898 PMC357049

[B19] Keleku-Lukwete N. Suzuki M. Yamamoto M. (2018). An overview of the advantages of KEAP1-NRF2 system activation during inflammatory disease treatment. Antioxid. Redox Signal 29 (17), 1746–1755. 10.1089/ars.2017.7358 28899203

[B20] Kensler T. W. Wakabayashi N. Biswal S. (2007). Cell survival responses to environmental stresses via the Keap1-Nrf2-ARE pathway. Annu. Rev. Pharmacol. Toxicol. 47, 89–116. 10.1146/annurev.pharmtox.46.120604.141046 16968214

[B21] Khanal P. Dey Y. N. Patil R. Chikhale R. Wanjari M. M. Gurav S. S. (2021). Combination of system biology to probe the anti-viral activity of andrographolide and its derivative against COVID-19. RSC Adv. 11 (9), 5065–5079. 10.1039/d0ra10529e 35424441 PMC8694486

[B22] Khor T. O. Huang M. T. Kwon K. H. Chan J. Y. Reddy B. S. Kong A. N. (2006). Nrf2-deficient mice have an increased susceptibility to dextran sulfate sodium-induced colitis. Cancer Res. 66 (24), 11580–11584. 10.1158/0008-5472.CAN-06-3562 17178849

[B23] Kobayashi M. Yamamoto M. (2006). Nrf2-Keap1 regulation of cellular defense mechanisms against electrophiles and reactive oxygen species. Adv. Enzyme Regul. 46, 113–140. 10.1016/j.advenzreg.2006.01.007 16887173

[B24] Kongpetch S. Kukongviriyapan V. Prawan A. Senggunprai L. Kukongviriyapan U. Buranrat B. (2012). Crucial role of heme oxygenase-1 on the sensitivity of cholangiocarcinoma cells to chemotherapeutic agents. PLoS One 7 (4), e34994. 10.1371/journal.pone.0034994 22514698 PMC3325916

[B25] Kucharzik T. Koletzko S. Kannengiesser K. Dignass A. (2020). Ulcerative colitis-diagnostic and therapeutic algorithms. Dtsch. Arztebl Int. 117 (33-34), 564–574. 10.3238/arztebl.2020.0564 33148393 PMC8171548

[B26] Kumar S. Singh B. Bajpai V. (2021). Andrographis paniculata (Burm.f.) Nees: traditional uses, phytochemistry, pharmacological properties and quality control/quality assurance. J. Ethnopharmacol. 275, 114054. 10.1016/j.jep.2021.114054 33831465

[B27] Lauridsen C. (2019). From oxidative stress to inflammation: redox balance and immune system. Poult. Sci. 98 (10), 4240–4246. 10.3382/ps/pey407 30371893

[B28] Le Berre C. Honap S. Peyrin-Biroulet L. (2023). Ulcerative colitis. Lancet 402 (10401), 571–584. 10.1016/S0140-6736(23)00966-2 37573077

[B29] Li X. Yuan W. Wu J. Zhen J. Sun Q. Yu M. (2022). Andrographolide, a natural anti-inflammatory agent: an Update. Front. Pharmacol. 13, 920435. 10.3389/fphar.2022.920435 36238575 PMC9551308

[B30] Liu J. Han X. Zhang T. Tian K. Li Z. Luo F. (2023). Reactive oxygen species (ROS) scavenging biomaterials for anti-inflammatory diseases: from mechanism to therapy. J. Hematol. Oncol. 16 (1), 116. 10.1186/s13045-023-01512-7 38037103 PMC10687997

[B31] Loboda A. Damulewicz M. Pyza E. Jozkowicz A. Dulak J. (2016). Role of Nrf2/HO-1 system in development, oxidative stress response and diseases: an evolutionarily conserved mechanism. Cell Mol. Life Sci. 73 (17), 3221–3247. 10.1007/s00018-016-2223-0 27100828 PMC4967105

[B32] Ma Q. (2013). Role of nrf2 in oxidative stress and toxicity. Annu. Rev. Pharmacol. Toxicol. 53, 401–426. 10.1146/annurev-pharmtox-011112-140320 23294312 PMC4680839

[B33] Mahmoud A. M. Germoush M. O. Al-Anazi K. M. Mahmoud A. H. Farah M. A. Allam A. A. (2018). Commiphora molmol protects against methotrexate-induced nephrotoxicity by up-regulating Nrf2/ARE/HO-1 signaling. Biomed. Pharmacother. 106, 499–509. 10.1016/j.biopha.2018.06.171 29990838

[B34] Osburn W. O. Karim B. Dolan P. M. Liu G. Yamamoto M. Huso D. L. (2007). Increased colonic inflammatory injury and formation of aberrant crypt foci in Nrf2-deficient mice upon dextran sulfate treatment. Int. J. Cancer 121 (9), 1883–1891. 10.1002/ijc.22943 17631644

[B35] Patil R. Jain V. (2021). Andrographolide: a review of analytical methods. J. Chromatogr. Sci. 59 (2), 191–203. 10.1093/chromsci/bmaa091 33221827

[B36] Paul G. Bataille F. Obermeier F. Bock J. Klebl F. Strauch U. (2005). Analysis of intestinal haem-oxygenase-1 (HO-1) in clinical and experimental colitis. Clin. Exp. Immunol. 140 (3), 547–555. 10.1111/j.1365-2249.2005.02775.x 15932518 PMC1809385

[B37] Peng S. Shen L. Yu X. Wu J. Zha L. Xia Y. (2023). miR-200a attenuated oxidative stress, inflammation, and apoptosis in dextran sulfate sodium-induced colitis through activation of Nrf2. Front. Immunol. 14, 1196065. 10.3389/fimmu.2023.1196065 37646040 PMC10461398

[B38] Peng S. Shen L. Yu X. Zhang L. Xu K. Xia Y. (2023). The role of Nrf2 in the pathogenesis and treatment of ulcerative colitis. Front. Immunol. 14, 1200111. 10.3389/fimmu.2023.1200111 37359553 PMC10285877

[B39] Pereira C. Grácio D. Teixeira J. P. Magro F. (2015). Oxidative stress and DNA damage: implications in inflammatory bowel disease. Inflamm. Bowel Dis. 21 (10), 2403–2417. 10.1097/MIB.0000000000000506 26193347

[B40] Poma P. (2020). NF-κB and disease. Int. J. Mol. Sci. 21 (23), 9181. 10.3390/ijms21239181 33276434 PMC7730361

[B41] Pu H. L. Chiang W. L. Maiti B. Liao Z. X. Ho Y. C. Shim M. S. (2014). Nanoparticles with dual responses to oxidative stress and reduced ph for drug release and anti-inflammatory applications. ACS Nano 8 (2), 1213–1221. 10.1021/nn4058787 24386907

[B42] Roessner A. Kuester D. Malfertheiner P. Schneider-Stock R. (2008). Oxidative stress in ulcerative colitis-associated carcinogenesis. Pathol. Res. Pract. 204 (7), 511–524. 10.1016/j.prp.2008.04.011 18571874

[B43] Schieber M. Chandel N. S. (2014). ROS function in redox signaling and oxidative stress. Curr. Biol. 24 (10), R453–R462. 10.1016/j.cub.2014.03.034 24845678 PMC4055301

[B44] Segal J. P. LeBlanc J. F. Hart A. L. (2021). Ulcerative colitis: an update. Clin. Med. (Lond). 21 (2), 135–139. 10.7861/clinmed.2021-0080 33762374 PMC8002778

[B45] Seyedian S. S. Nokhostin F. Malamir M. D. (2019). A review of the diagnosis, prevention, and treatment methods of inflammatory bowel disease. J. Med. Life 12 (2), 113–122. 10.25122/jml-2018-0075 31406511 PMC6685307

[B46] Srivastava S. K. Yadav U. C. Reddy A. B. Saxena A. Tammali R. Shoeb M. (2011). Aldose reductase inhibition suppresses oxidative stress-induced inflammatory disorders. Chem. Biol. Interact. 191 (1-3), 330–338. 10.1016/j.cbi.2011.02.023 21354119 PMC3103634

[B47] Tundis R. Patra J. K. Bonesi M. Das S. Nath R. Das Talukdar A. (2023). Anti-Cancer agent: the labdane diterpenoid-andrographolide. Plants (Basel) 12 (10), 1969. 10.3390/plants12101969 37653887 PMC10221142

[B48] Wanandi S. I. Limanto A. Yunita E. Syahrani R. A. Louisa M. Wibowo A. E. (2020). *In silico* and *in vitro* studies on the anti-cancer activity of andrographolide targeting survivin in human breast cancer stem cells. PLoS One 15 (11), e0240020. 10.1371/journal.pone.0240020 33211707 PMC7676700

[B49] Wang L. Zhang X. Xiong X. Zhu H. Chen R. Zhang S. (2022). Nrf2 regulates oxidative stress and its role in cerebral ischemic stroke. Antioxidants (Basel) 11 (12), 2377. 10.3390/antiox11122377 36552584 PMC9774301

[B50] Zeng X. Yang M. Ye T. Feng J. Xu X. Yang H. (2023). Mitochondrial GRIM-19 loss in parietal cells promotes spasmolytic polypeptide-expressing metaplasia through NLR family pyrin domain-containing 3 (NLRP3)-mediated IL-33 activation via a reactive oxygen species (ROS) -NRF2- Heme oxygenase-1(HO-1)-NF-кB axis. Free Radic. Biol. Med. 202, 46–61. 10.1016/j.freeradbiomed.2023.03.024 36990300

[B51] Zheng X. Qiu J. Zhang H. Gao N. Jiang T. Gong Y. (2023). PD184352 exerts anti-inflammatory and antioxidant effects by promoting activation of the Nrf2/HO-1 axis. Biochem. Pharmacol. 211, 115542. 10.1016/j.bcp.2023.115542 37028460

[B52] Zhu Q. Zheng P. Chen X. Zhou F. He Q. Yang Y. (2018). Andrographolide presents therapeutic effect on ulcerative colitis through the inhibition of IL-23/IL-17 axis. Am. J. Transl. Res. 10 (2), 465–473. 29511440 PMC5835811

